# Temporal Transitions in Dietary Patterns and Their Associations with Overweight and Obesity Among Middle-Aged and Older Adults in Southern China: Evidence from Four Rounds of Nutrition Surveillance, 2002–2022

**DOI:** 10.3390/nu18193222

**Published:** 2026-09-29

**Authors:** Shuaijing Zhang, Yunkai Lin, Jianhang Li, Jiewen Peng, Jian Wen, Zihui Chen, Qi Jiang, Panpan Huang, Xingfen Yang, Guiyuan Ji, Wei Wu

**Affiliations:** 1 NMPA Key Laboratory for Safety Evaluation of Cosmetics, Guangdong Provincial Key Laboratory of Tropical Disease Research, School of Public Health, Southern Medical University, Guangzhou 510515, China; 2 Guangdong Provincial Institute of Public Health, Guangdong Provincial Center for Disease Control and Prevention, Guangzhou 511400, China; 3 School of Public Health, Sun Yat-Sen University, Guangzhou 510080, China; 4 Guangdong Provincial Center for Disease Control and Prevention, Guangzhou 511400, China

**Keywords:** dietary pattern, obesity, overweight, central obesity, southern China, middle-aged and older adults

## Abstract

Background: China has experienced substantial dietary transitions over recent decades, accompanied by increasing burdens of overweight and obesity. However, long-term changes in dietary patterns and their associations with obesity among middle-aged and older adults remain insufficiently characterized. Methods: We analyzed data from 8502 adults aged ≥45 years participating in four waves (2002–2022) of the China National Nutrition Surveys in Guangdong Province. Dietary patterns were identified using k-means cluster analysis based on 23 food groups. Temporal changes in dietary pattern distributions were assessed using Rao–Scott adjusted chi-square tests and survey-weighted logistic regression models incorporating linear and quadratic terms for survey year. Multivariable logistic regression models were used to examine associations between dietary patterns and overweight, obesity, and central obesity. Results: Three dietary patterns were identified: a traditional cereal-based pattern, a transitional mixed pattern, and an animal-rich diverse pattern. The prevalence of the Traditional Cereal-based pattern decreased from 15.8% in 2002 to 0.4% in 2022, whereas the Animal-rich Diverse pattern increased from 19.2% to 47.0%. Trend analyses confirmed significant temporal changes in all dietary patterns after false discovery rate correction. Compared with the Traditional Cereal-based Pattern, the Transitional Mixed Pattern and Animal-rich Diverse Pattern was associated with higher odds of overweight/obesity, with adjusted ORs of 1.57 (95% CI: 1.29–1.90) and 1.42 (95% CI: 1.14–1.77), respectively. Similar associations were observed for central obesity, with corresponding adjusted ORs of 1.54 (95% CI: 1.26–1.89) and 1.35 (95% CI: 1.08–1.71), respectively. Conclusions: Significant dietary transitions occurred among middle-aged and older adults in Guangdong Province from 2002 to 2022. Dietary patterns characterized by higher intakes of animal-source and energy-dense foods were associated with greater odds of overweight/obesity and central obesity. These findings suggest that dietary diversity alone may not necessarily represent a healthier dietary pattern during nutrition transition and highlight the potential importance of considering overall dietary composition and energy balance in obesity prevention strategies.

## 1. Introduction

Overweight and obesity remain among the most pressing global public health challenges, with rapidly increasing prevalence, particularly in low- and middle-income countries undergoing rapid socioeconomic and nutritional transitions [1]. China has experienced a substantial increase in the prevalence of overweight and obesity over recent decades. According to the latest national estimates for 2015–2019 based on Chinese BMI criteria, 34.3% of Chinese adults aged ≥18 years were overweight and 16.4% were obese [2]. Nationally representative survey data further demonstrated that, from 2002 to 2018, the prevalence of obesity increased from 7.1% to 16.4%, while the combined prevalence of overweight and obesity increased from 29.9% to 50.7% [3]. These trends have occurred alongside profound changes in dietary behaviours driven by rapid urbanization, economic development, and population ageing [2,4,5]. Middle-aged and older individuals are particularly vulnerable because age-related changes in body composition and energy metabolism, together with the accumulation of chronic conditions, may increase susceptibility to excessive adiposity and its associated health consequences [1,6,7]. Identifying dietary patterns and their associations with obesity in this population is therefore important for informing targeted nutritional interventions and healthy ageing strategies.

Dietary pattern analysis provides a useful framework for examining the role of diet in obesity because it considers habitual combinations of food consumption rather than isolated foods or nutrients [8]. By capturing the synergistic and cumulative effects of the overall diet, dietary patterns may better represent real-world eating behaviours and the cultural, social, and environmental contexts in which food choices occur [1,9]. Consequently, dietary pattern analysis has become an increasingly important approach for investigating diet-related chronic diseases and informing population-based dietary recommendations. Methodologically, dietary pattern analysis can be broadly classified into a priori and a posteriori approaches [10]. Although a priori dietary indices facilitate comparisons across populations, they may not adequately capture regional dietary diversity. In contrast, a posteriori data-driven methods, such as cluster analysis, identify empirically derived dietary patterns that better reflect local food consumption behaviours and cultural heterogeneity [11].

Previous population-based studies have identified marked regional and temporal heterogeneity in dietary patterns across China. Nationwide evidence has demonstrated clear regional variation in dietary patterns, with rice-based dietary patterns predominating in southern China and wheat-based patterns being more characteristic of northern regions [12]. Other population-based studies have similarly identified traditional southern and northern dietary patterns, together with a Western dietary pattern characterized by greater consumption of animal-sourced foods, dairy products, and other non-traditional foods [13,14]. In addition to this regional heterogeneity, dietary patterns have evolved alongside China’s rapid socioeconomic development and nutrition transition. Long-term evidence from the China Health and Nutrition Survey showed a gradual decline in the traditional southern pattern from 1991 to 2015, accompanied by increasing adherence to modern and meat-based patterns, reflecting the ongoing nutrition transition in China [3].

Within this broader transition, examining dietary patterns within specific regions may provide insights that are not fully captured by national-level analyses. Guangdong Province, located in southern coastal China, represents a unique nutritional and cultural setting. Traditional Cantonese diets are characterized by relatively high consumption of aquatic foods, fresh vegetables, soup-based dishes, and diverse food combinations shaped by long-standing culinary traditions [15,16]. At the same time, rapid economic growth and urbanization over recent decades have substantially altered food availability and dietary behaviours in Guangdong. The coexistence of distinctive traditional dietary practices and rapid nutrition transition raises the possibility that dietary patterns in this population have undergone substantial changes over time. However, evidence characterizing long-term changes in empirically derived dietary patterns among middle-aged and older adults in southern coastal China remains limited.

Previous studies have linked empirically derived dietary patterns with obesity-related outcomes in Chinese populations, including higher obesity risk associated with some modern or animal-food-rich dietary patterns [1,17]. However, existing evidence has predominantly focused on associations observed at a single time point or in general adult populations, whereas less is known about how population-level dietary patterns have transitioned over extended periods and whether the patterns accompanying this transition are associated with obesity among middle-aged and older adults. Moreover, central obesity is highly prevalent in middle-aged and older adults and is strongly associated with cardiometabolic disorders [18,19], further supporting the need to consider both overall and abdominal adiposity.

To address these knowledge gaps, the present study used data from multiple rounds of nutritional surveillance conducted in Guangdong Province between 2002 and 2022 to identify empirically derived dietary patterns among adults aged 45 years. We aimed to characterize temporal transitions in these dietary patterns over the two-decade period and to evaluate their associations with overweight/obesity and central obesity. By integrating long-term dietary surveillance with obesity-related outcomes, this study sought to provide region-specific evidence on the health implications accompanying China’s ongoing nutrition transition.

## 2. Materials and Methods

### 2.1. Study Design and Subjects

**Figure 1:**
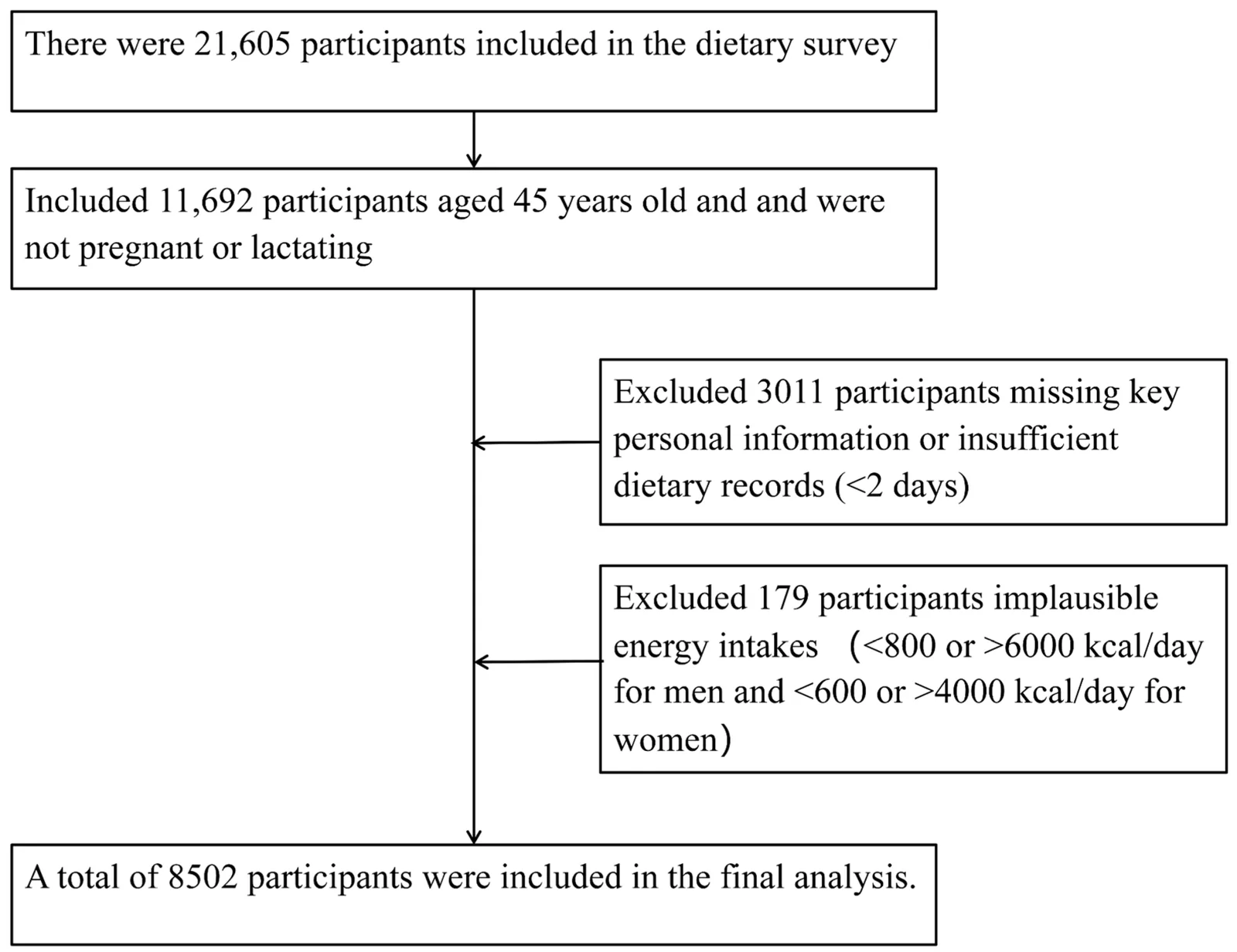
Study population flow chart.

The present study was based on four rounds of the China National Nutrition Surveys (CNNS) in 2002, 2012, 2015, and 2022. The CNNS is a nationally representative survey program implemented by the Chinese Center for Disease Control and Prevention using multistage stratified cluster sampling methods. Detailed information regarding the survey design and procedures has been described elsewhere [20,21]. Given the substantial demographic, socioeconomic, and dietary transitions experienced in Guangdong Province during recent decades, and the availability of comparable dietary and health information across multiple CNNS waves, data from Guangdong Province were selected for the present analysis. The study population selection process is presented in Figure 1.

### 2.2. Data Collection and Dietary Assessment

The present study used data from Guangdong Province collected within the framework of the CNNS. In the original CNNS surveys, demographic, socioeconomic, lifestyle, and dietary information was collected according to standardized survey protocols by trained investigators. Demographic, socioeconomic, and lifestyle information was collected through face-to-face household interviews, with respondents providing the information personally.

Dietary intake was assessed using three consecutive 24 h dietary recalls combined with a household food weighing method. Participants reported all foods and beverages consumed during the previous 24 h for each recall day, including two weekdays and one weekend day [22]. Household food inventories were weighed at the beginning and end of each survey day to improve accuracy, and culinary condiments were assessed separately using standardized weighing procedures.

Dietary records from Guangdong Province were processed using R software (version 4.5.1). Individual food items were linked through the CNNS food coding system to the corresponding nutrient composition information in the Chinese Food Composition Tables [23,24,25]. Daily energy and nutrient intakes were calculated based on the amount of each food consumed and its corresponding nutrient composition. The average intake across the three dietary recall days was subsequently calculated for each participant.

### 2.3. Dietary Pattern Identification

Individual food items were classified into 23 predefined food groups according to the existing CNNS food coding system and the food classification information provided in the Chinese Food Composition Tables [21,22,23], with consideration of similarities in nutritional composition and culinary use. Where applicable, recorded food weights were standardized to a comparable edible-weight basis using appropriate food-specific conversion factors before food-group aggregation. A prespecified food-group mapping scheme was then applied programmatically using R software (version 4.5.1), and food items assigned to the same food group were aggregated to obtain individual daily food-group intakes. The food-group classification and processing framework was harmonized across survey waves to ensure comparability. Detailed definitions and representative foods for the 23 food groups are provided in Supplementary Table S8.

To improve comparability across individuals with different physiological requirements, food-group intakes were standardized using a standard person coefficient based on age, sex, physiological status, and physical activity level, following the approach used in previous China Nutrition and Health Surveillance studies [26]. Specifically, individual food intakes were divided by the corresponding standard person coefficient to generate standardized intake values expressed relative to a reference adult equivalent. Energy-adjusted food intakes were subsequently standardized as z-scores prior to clustering analysis.

To identify dietary patterns, standardized daily intakes of the 23 food groups were subjected to cluster analysis. Several clustering algorithms, including K-means and hierarchical clustering using Ward’s and average-linkage methods, were evaluated. Candidate solutions with two to five clusters (k = 2–5) were compared using multiple internal validity indices, including the silhouette coefficient, Calinski–Harabasz (CH) index, Davies–Bouldin (DB) index, and Dunn index [27]. Cluster size distribution and nutritional interpretability were also considered. The final clustering solution was selected through a comprehensive evaluation of internal validity, cluster size distribution, and nutritional interpretability, rather than relying on any single statistical criterion. Based on this evaluation, the three-cluster K-means solution was retained for the primary analyses, as it yielded three adequately sized and nutritionally interpretable dietary patterns [28]. Detailed results are presented in Figure S1 and Table S1.

The resulting dietary patterns were characterized according to their food-group intake profiles and were labeled as the Traditional Cereal-based pattern, Transitional Mixed pattern, and Animal-rich Diverse pattern. The dietary characteristics of the three patterns are presented in Supplementary Table S2.

To evaluate clustering stability, we performed resampling-based validation and calculated the Adjusted Rand Index (ARI) between the original and resampled clustering solutions [29]. Higher ARI values indicated greater similarity between the original and resampled clustering solutions and therefore greater clustering stability.

### 2.4. Outcome

All anthropometric measurements were performed by trained staff following standardized protocols. Body mass index (BMI) was calculated as weight in kilograms divided by height in meters squared (kg/m^2^). For adults aged 45–79 years, overweight was defined as BMI of 24.0–27.9 kg/m^2^, and obesity as BMI of ≥28.0 kg/m^2^. Central obesity was defined as waist circumference ≥ 90 cm in men and ≥85 cm in women [30]. For participants aged 80 years or older, age-specific BMI criteria (22.0–26.9 kg/m^2^) were applied [31,32]. According to the Chinese criteria for adult overweight and obesity classification, both BMI and waist circumference were used to classify obesity status [32].

### 2.5. Covariates

Baseline covariates included residential area, smoking status, alcohol consumption, educational attainment, physical activity, occupational status, income level, and self-reported history of chronic diseases. The residential area was categorized as urban or rural. Smoking status was classified as smoker, non-smoker, or unknown. Alcohol consumption was categorized as drinker or non-drinker. Educational attainment was grouped into elementary (illiterate and primary school), secondary (junior high school, high school, and vocational college), and higher education levels (university and above). Physical activity was classified as low, moderate, or high according to occupational activity intensity.

Occupational status was classified into six categories: student, retired or unemployed, office worker, business service personnel, manual labor, and others. Income level was defined according to OECD criteria, with poverty defined as household disposable income below 50% of the median income for each survey yea [17]. Self-reported chronic diseases were defined as physician-diagnosed hypertension, diabetes, or dyslipidemia reported by participants during the household interview.

### 2.6. Data Analysis

Baseline characteristics of participants were summarized according to dietary pattern groups and were presented as means (standard deviations) or medians (interquartile ranges) for continuous variables and as counts (percentages) for categorical variables.

Associations between dietary patterns and health outcomes were examined using multivariable logistic regression models, with results presented as odds ratios (ORs) and 95% confidence intervals (CIs). Overweight/obesity and central obesity were treated as dependent variables, and dietary patterns were included as the primary exposure. Survey wave was included as a categorical covariate in all regression models. Four logistic regression models were constructed. Model 1 was an unadjusted model. Model 2 was adjusted for age, sex, survey year, and region. Model 3 was further adjusted for education level and income level. Model 4 was additionally adjusted for smoking status, alcohol consumption, physical activity, and total energy intake. Total energy intake was included as a continuous covariate in the logistic regression models.

To examine changes in dietary patterns over survey years, we used Rao–Scott chi-square tests to compare the overall distribution across years. For each pattern, survey-weighted logistic regression with linear and quadratic year terms (centered at 2012) was applied; joint Wald tests assessed overall significance, and *p*-values were adjusted for multiple testing using the false discovery rate.

Potential effect modification was assessed by including interaction terms between dietary patterns and sex, age, residential area, and survey year. Stratified analyses were subsequently conducted. Sensitivity analyses were performed by excluding participants with chronic diseases. All statistical analyses were conducted using R software (version 4.5.1), and a two-sided *p* < 0.05 was considered statistically significant.

## 3. Results

### 3.1. Population Characteristics of Dietary Patterns

Baseline characteristics are summarized in Table 1. Participants differed significantly across dietary patterns in sociodemographic, lifestyle, and health-related characteristics.

Compared with participants in the Traditional Cereal-based pattern, those in the Transitional Mixed and Animal-rich Diverse patterns were more likely to be younger, urban residents, and individuals with higher educational attainment and household income. Participants in the Traditional Cereal-based pattern showed higher levels of physical activity and were more likely to have rural residence, whereas participants in the Animal-rich Diverse pattern had the highest proportion of urban residence and favorable socioeconomic characteristics.

### 3.2. Identification of Dietary Patterns

Among the 8502 participants included in the four survey waves from 2002 to 2022, three distinct dietary patterns were identified using K-means cluster analysis. The three patterns were labeled as the Traditional Cereal-based Pattern, Transitional Mixed Pattern, and Animal-rich Diverse Pattern according to their characteristic food group distributions. The clustering solution demonstrated good reproducibility, with a mean ARI of 0.86 (SD = 0.16), ranging from 0.54 to 0.99. These findings indicate substantial agreement between the original and resampled clustering results, suggesting good stability of the identified dietary patterns.

**Figure 2:**
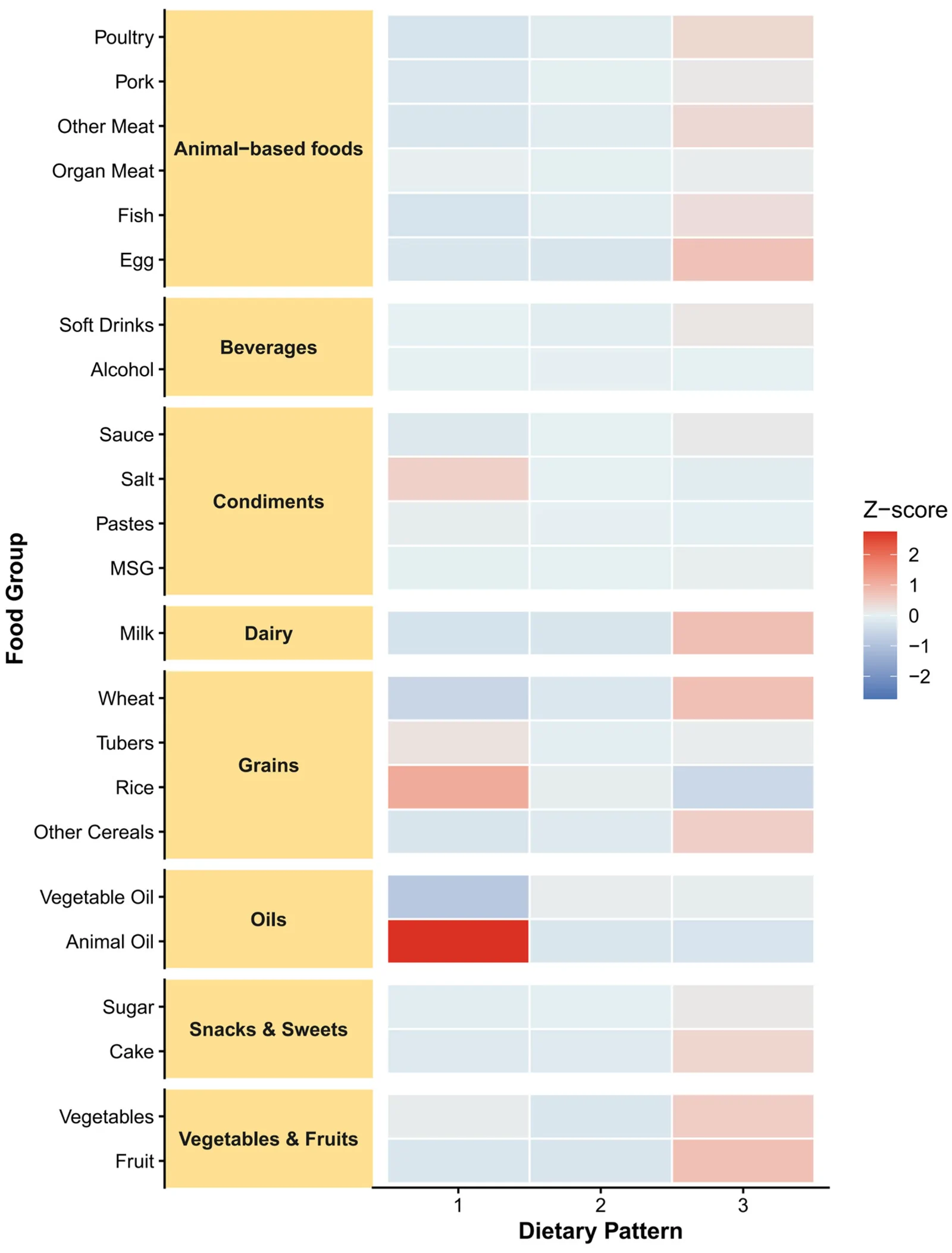
Z-score heatmap of food group intake across three dietary patterns.

The dietary patterns were characterized as follows: Pattern 1 was labeled the Traditional Cereal-based pattern and was characterized by higher consumption of rice and rice products, animal oil, and salt, with relatively lower consumption of plant oil, aquatic products, dairy, meat, and fruits. Pattern 2 was labeled the Transitional Mixed pattern and showed intermediate intake levels across most food groups, with relatively higher consumption of rice and plant oil and lower intake of fruits, vegetables, dairy products, and aquatic products. Pattern 3 was labeled the Animal-rich Diverse pattern and was characterized by higher consumption of animal-derived foods (eggs, dairy products, fish, and meat) and fruits/vegetables, together with lower intake of rice products and animal oil.The standardized food-group intake profiles across the three dietary patterns are visualized in Figure 2.

**Figure 3:**
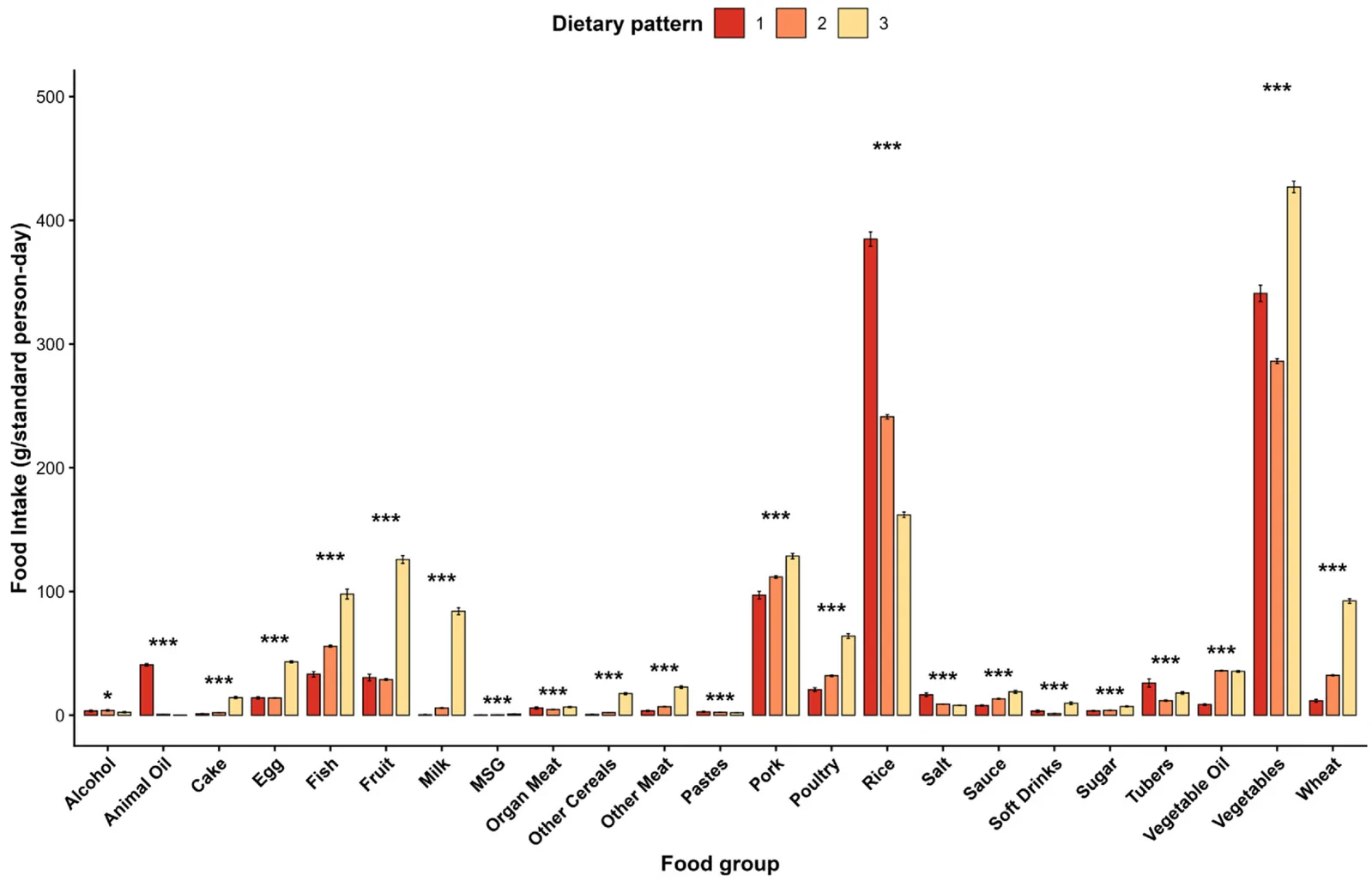
Food group intake across dietary patterns and the overall population. * *p* < 0.05, ** *p* < 0.01, *** *p* < 0.001.

Absolute food intake analyses further supported these distinctions among dietary patterns (Figure 3 and Table S2). Nutrient profiles also differed across patterns. Pattern 1 showed the highest carbohydrate intake (344.79 g/day), whereas Pattern 3 had the highest protein and fat intake (107.00 g/day and 104.54 g/day, respectively). Details of the nutrient intakes for each dietary pattern are presented in Table S3.

### 3.3. Temporal Changes in Dietary Patterns

The distribution of dietary patterns differed significantly across survey waves (Rao–Scott adjusted χ^2^ test, *p* < 0.001). The prevalence of the Traditional Cereal-based pattern declined markedly from 15.8% in 2002 to 0.4% in 2022, whereas the Animal-rich Diverse pattern increased from 19.2% to 47.0%. The Transitional Mixed pattern remained the predominant dietary pattern throughout the study period but showed substantial fluctuations across survey waves (Figure S3). In survey-weighted logistic regression models incorporating both linear and quadratic terms of survey year, significant temporal trends were observed for the prevalence of all three dietary patterns after FDR correction (all adjusted joint Wald, *p* < 0.001). The prevalence of the Traditional Cereal-based Pattern showed a significant declining trend over time (linear OR = 0.814, 95% CI: 0.773–0.857), whereas the prevalence of the Animal-rich Diverse Pattern increased progressively (linear OR = 1.065, 95% CI: 1.055–1.074). In contrast, the Transitional Mixed Pattern demonstrated a significant nonlinear temporal trend, as indicated by the significant quadratic term, suggesting variation across survey waves rather than a consistent directional change (Table 2).

### 3.4. Associations Between Dietary Patterns and Overweight/Obesity

**Figure 4:**
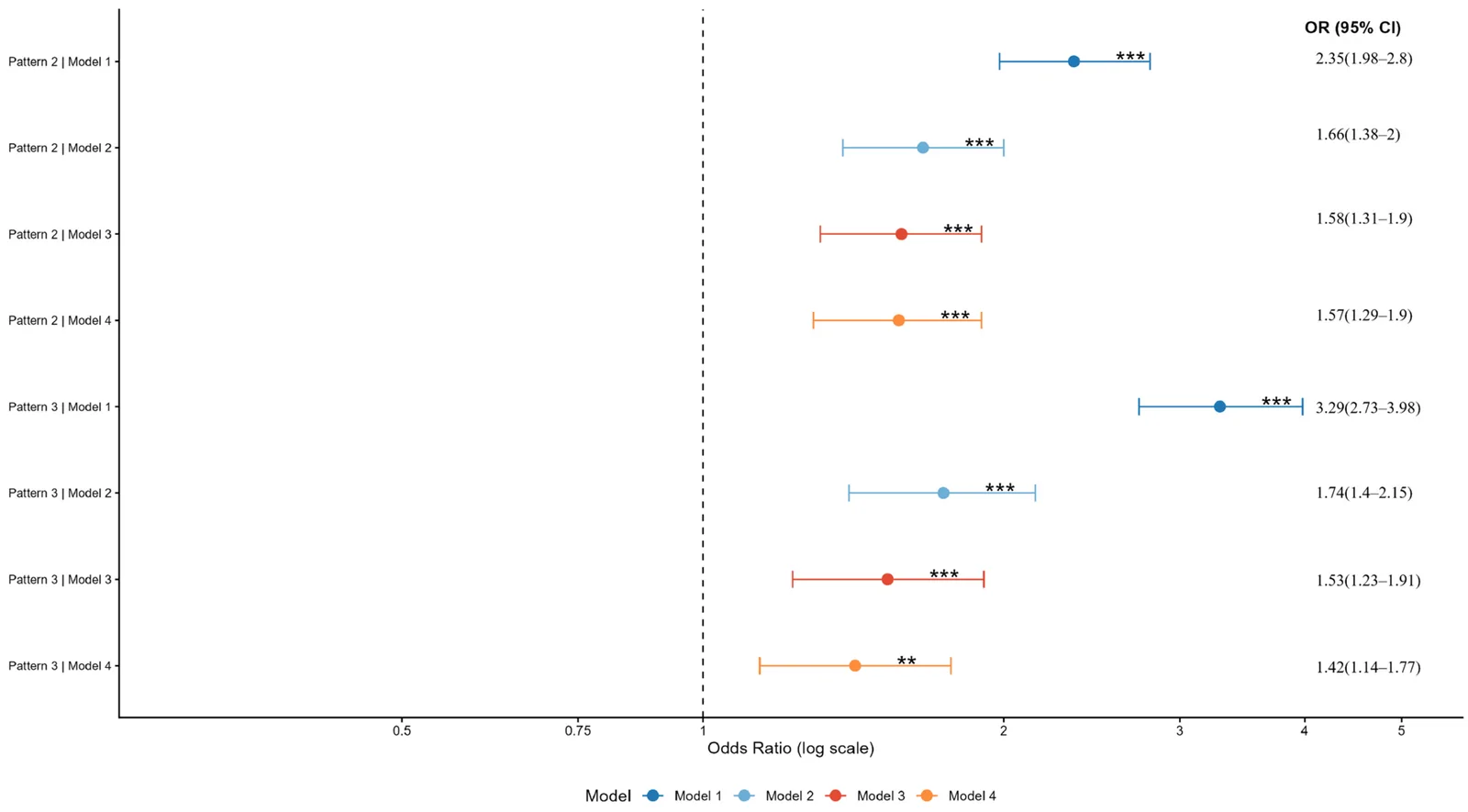
Overweight/Obesity forest. Model 1 was unadjusted. Model 2 was adjusted for age, sex, survey year, and region. Model 3 was further adjusted for education level and income level. Model 4 was additionally adjusted for smoking status, alcohol consumption, physical activity, and total energy intake. * *p* < 0.05, ** *p* < 0.01, *** *p* < 0.001.

Multivariable logistic regression models were used to examine associations between dietary patterns and overweight/obesity. Compared with the traditional cereal-based pattern (Pattern 1), both the transitional mixed pattern (Pattern 2) and the animal-rich diverse pattern (Pattern 3) were associated with higher odds of overweight and obesity after adjustment for potential confounders. In the fully adjusted model, the odds ratios for overweight/obesity were 1.57 (95% CI: 1.29–1.90) for the Transitional Mixed pattern and 1.42 (95% CI: 1.14–1.77) for the Animal-rich Diverse pattern (Figure 4). The associations remained generally consistent across models with incremental adjustment for sociodemographic and lifestyle factors. Detailed results of all regression models are presented in Table S4.

### 3.5. Associations with Overweight, Obesity, and Central Obesity, Including Subgroup and Interaction Analyses

We further examined associations with each obesity phenotype separately, including overweight, obesity, and central obesity, with results presented in Table S5. Compared with the Traditional Cereal-based pattern, the Transitional Mixed pattern was positively associated with overweight, obesity, and central obesity after multivariable adjustment, whereas the Animal-rich Diverse pattern was positively associated with obesity and central obesity but not significantly associated with overweight. For overweight, the adjusted ORs were 1.41 (95% CI: 1.11–1.80; *p* = 0.005) for the Transitional Mixed pattern and 1.26 (95% CI: 0.96–1.66; *p* = 0.098) for the Animal-rich Diverse pattern. For obesity, the corresponding ORs were 1.73 (95% CI: 1.35–2.23; *p* < 0.001) and 1.58 (95% CI: 1.19–2.10; *p* = 0.001), respectively. For central obesity, the adjusted ORs were 1.54 (95% CI: 1.26–1.89; *p* < 0.001) and 1.35 (95% CI: 1.08–1.71; *p* = 0.01).

Significant effect modification was observed for sex (*p* for interaction = 0.005) and survey year (*p* for interaction = 0.013), whereas no significant interactions were observed for age or residential area. In analyses stratified by residential area, the associations with overweight/obesity were generally positive in both rural and urban participants, with no statistically significant interaction by residential area (*p* for interaction = 0.239). Detailed subgroup estimates are presented in Table S6.

### 3.6. Sensitivity Analyses

In sensitivity analyses excluding participants with hypertension, diabetes, or dyslipidemia, the associations between dietary patterns and overweight/obesity were attenuated and no longer statistically significant (Table S7). These findings suggest that the observed associations may partly reflect the influence of underlying chronic conditions or related behavioral changes. Given the cross-sectional nature of the study, residual confounding and reverse causation cannot be completely excluded.

## 4. Discussion

This study provides region-specific evidence that three distinct dietary patterns were identified among middle-aged and older adults in Guangdong Province, with both the transitional mixed and animal-rich diverse patterns being associated with a higher likelihood of obesity. Compared with the traditional cereal-based dietary pattern, both the transitional mixed pattern and the animal-rich diverse pattern were associated with increased odds of obesity outcomes. Importantly, these dietary patterns were also closely linked with socioeconomic and lifestyle characteristics during nutrition transition, with the Traditional Cereal-based Pattern being more common among rural populations and individuals with lower socioeconomic status, whereas the Animal-rich Diverse Pattern was more prevalent among urban residents with higher socioeconomic status and lower physical activity levels. These findings suggest that dietary patterns should be interpreted not only as combinations of food consumption but also as broader behavioral and social contexts that may shape obesity-related outcomes. Furthermore, dietary diversity alone may not necessarily indicate higher dietary quality, particularly in populations undergoing rapid nutrition transition, where increases in food variety may occur alongside greater consumption of energy-dense foods.

The dietary patterns identified in this study are broadly consistent with those reported in previous studies in Chinese populations, while also reflecting region-specific characteristics and the ongoing process of nutrition transition. The traditional cereal-based pattern resembled the conventional southern dietary pattern, characterized by high consumption of rice and relatively low intake of animal-source foods, and was more prevalent among rural and socioeconomically disadvantaged populations [12,33]. In contrast, the Animal-rich Diverse Pattern shared similarities with modern dietary patterns reported in previous studies, including greater consumption of meat, aquatic products, dairy products, and other energy-contributing foods. However, unlike a typical Western dietary pattern, this pattern also included relatively higher consumption of fruits and vegetables, indicating a diversified but potentially energy-dense dietary structure [12,34]. The Transitional Mixed Pattern did not correspond directly to a previously established dietary pattern but appeared to represent an intermediate dietary profile characterized by a relatively balanced distribution across food groups [11]. Although similar “prudent” dietary patterns in high-income populations are often associated with favorable metabolic profiles [11,35], the present findings suggest that the health implications of dietary patterns may depend on the local dietary environment, socioeconomic context, and stage of nutrition transition.

The Traditional Cereal-based Pattern identified in this study showed characteristics consistent with traditional dietary practices in southern China, including a high contribution of staple cereals and relatively lower consumption of animal-source foods. Historically, such dietary patterns have been considered representative of traditional Chinese diets, which often emphasize cereal-based meals accompanied by vegetables and moderate amounts of animal-source foods. However, the association between this dietary pattern and obesity-related outcomes should be interpreted cautiously, as the lower prevalence of obesity observed among individuals following this pattern may partly reflect differences in socioeconomic status, residential environment, physical activity, and other lifestyle factors rather than dietary composition alone. Therefore, this pattern may represent not only a traditional dietary structure but also a broader lifestyle profile associated with earlier stages of nutrition transition. When compared with the traditional Lingnan dietary pattern, which emphasizes high intake of vegetables and aquatic foods, moderate consumption of animal products, and low levels of salt and oil [36], the transitional mixed and the animal-rich diverse did not fully reflect these traditional dietary characteristics. Instead, these patterns may represent dietary structures emerging during ongoing nutrition transition, characterized by increased dietary diversity accompanied by greater consumption of animal-source foods and energy-dense dietary components. These findings suggest that dietary patterns perceived as more diverse or nutritionally improved should not necessarily be interpreted as healthier dietary patterns. In populations undergoing rapid nutrition transition, changes in lifestyle behaviors, and reduced physical activity, which may jointly contribute to obesity-related outcomes [34].

The Animal-rich Diverse Pattern was positively associated with the combined overweight/obesity outcome and central obesity in the primary analyses, while the phenotype-specific analysis showed a significant association with obesity but not with overweight. Several potential explanations may account for these associations. First, this dietary pattern was characterized by higher consumption of animal-source foods and energy-contributing food groups, which may be associated with greater overall energy intake and dietary energy density. Although energy intake was not directly evaluated in the present study, previous evidence suggests that dietary patterns characterized by higher energy density may be linked with increased susceptibility to excessive energy balance and adiposity [37,38,39,40,41,42]. Importantly, the observed association should not be interpreted as an adverse effect of animal-source foods alone. Animal-source foods represent a heterogeneous group, including foods with different nutritional profiles and potential metabolic effects. Therefore, the association observed in this study likely reflects the overall dietary composition, including the balance between animal-source foods, plant-based foods, and other dietary components within the pattern. Furthermore, this dietary pattern may represent a broader lifestyle transition accompanying socioeconomic development, including changes in food availability, dietary behaviors, and physical activity patterns. These accompanying lifestyle changes may partly contribute to the observed association between the Animal-rich Diverse Pattern and obesity-related outcomes [42].

The Transitional Mixed Pattern also showed positive associations with obesity-related outcomes in the primary analyses. This pattern may represent an intermediate dietary profile during nutrition transition, characterized by simultaneous retention of traditional dietary components and increasing consumption of diverse food groups. The association between this pattern and obesity may reflect the complex interaction between dietary changes and accompanying lifestyle transitions rather than the effect of a single dietary component. As dietary behaviors evolve, changes in physical activity, sedentary behavior, and socioeconomic circumstances may occur concurrently, making it difficult to separate the independent contribution of individual dietary patterns in cross-sectional analyses [43,44,45,46,47,48,49]. The association observed for the Transitional Mixed Pattern should also be interpreted cautiously. In sensitivity analyses, the positive associations were substantially attenuated and no longer statistically significant after excluding participants with hypertension, diabetes, or dyslipidemia (Table S7). Similar observations have been reported in previous epidemiological studies, suggesting that disease-related dietary modifications and other behavioral changes following chronic disease diagnosis may partly influence the observed associations [50,51].

Subgroup analyses further indicated that the associations between dietary patterns and obesity were not uniform across populations. Sex-stratified analyses indicated that the associations between dietary patterns and obesity-related outcomes were not completely consistent between men and women. The Animal-rich Diverse Pattern showed significant associations with obesity indicators in both sexes, although some differences in effect estimates were observed. These findings suggest that sex-related factors may modify the relationship between dietary patterns and obesity; however, the underlying mechanisms remain unclear. Differences in lifestyle behaviors, energy expenditure, body composition, and socioeconomic circumstances may partly contribute to the observed heterogeneity. Further studies incorporating biological and behavioral measurements are needed to clarify these potential sex-specific pathways [52,53].

Residential area was also considered because the dietary patterns differed markedly in their urban–rural distribution. The Traditional Cereal-based Pattern was predominantly observed among rural participants, whereas the Animal-rich Diverse Pattern was more common among urban participants, raising the possibility that residential context might partly account for the observed associations with obesity. However, residence-stratified analyses showed broadly similar directions of association with overweight/obesity among rural and urban participants, and no statistically significant interaction between dietary pattern and residential area was observed (*p* for interaction = 0.239; Table S6). These findings suggest that urban–rural residence alone is unlikely to fully explain the observed associations. Nevertheless, the marked differences in pattern distribution by residence indicate that dietary patterns may partly reflect broader socioeconomic, environmental, and lifestyle characteristics associated with urbanization, and residual confounding by these factors cannot be completely excluded.

The interaction analysis between dietary patterns and survey years suggested that the associations between dietary patterns and obesity outcomes may have varied across different stages of the nutrition transition. This temporal heterogeneity may reflect changes in the broader social, economic, and behavioral environment over the two decades of observation [54]. As dietary environments evolve, the relationship between specific dietary patterns and obesity may also change due to concurrent changes in food availability, physical activity patterns, and population characteristics. These findings highlight the importance of interpreting dietary patterns within their historical and contextual background rather than considering them as static exposures [55].

From a public health perspective, the findings emphasize that dietary assessment during nutrition transition should consider overall dietary composition rather than dietary diversity alone. Although increasing food variety is generally encouraged, greater diversity accompanied by higher consumption of energy-dense foods may not necessarily correspond to healthier dietary patterns. Public health strategies should therefore focus on promoting balanced dietary structures, appropriate energy intake, and sustainable lifestyle behaviors, while considering the socioeconomic and cultural context of different populations.

This study has several strengths. First, it used four waves of nutrition surveillance data spanning two decades, allowing for an evaluation of temporal changes in dietary pattern distributions during a period of rapid nutrition transition. Second, dietary intake was assessed using three consecutive 24 h dietary recalls, which may still be subject to day-to-day variation and recall bias and may not fully capture long-term habitual dietary intake. Third, the use of multiple cluster validation indicators and adjustment for a range of demographic, socioeconomic, and lifestyle factors strengthened the robustness of the analyses. Finally, this study provides region-specific evidence from Guangdong Province, contributing to a more contextually grounded understanding of diet–obesity relationships in a population undergoing rapid nutrition transition.

Several limitations should be considered. First, the cross-sectional nature of each survey wave prevents causal inference, and the observed associations should not be interpreted as evidence that specific dietary patterns directly cause obesity. Second, dietary intake was assessed using three consecutive 24 h dietary recallsl, which may introduce measurement error due to day-to-day variation and recall bias. Third, although extensive covariates were adjusted for, residual confounding from unmeasured factors, such as genetic susceptibility and detailed dietary behaviors, cannot be completely excluded. Finally, dietary patterns were derived using cluster analysis, and the identified patterns may depend on the selected food groups and analytical decisions.

## 5. Conclusions

In conclusion, this study identified three distinct dietary patterns among middle-aged and older adults in southern China, which were differentially associated with overweight/obesity and central obesity. Dietary patterns characterized by higher intake of animal-sourced foods and energy-dense foods were associated with higher odds of obesity. These findings highlight the potential importance of considering overall dietary structure, rather than dietary diversity alone, in the context of ongoing nutrition transition. From a public health perspective, attention should be given to the potential associations between increasingly energy-dense dietary patterns and obesity-related outcomes, particularly among socioeconomically advantaged populations. Future longitudinal studies are needed to further clarify the causal relationships underlying these associations.

## Figures and Tables

**Table 1:** Characteristics across dietary patterns (*n* = 8502).

Characteristics	Traditional Cereal-Based Pattern *n* = 681	Transitional Mixed Pattern *n* = 5748	Animal-Rich Diverse Patter *n* = 2073	Total *n* = 8502	*p* Value
Sex (%)					<0.001
Male	358 (52.6)	2833 (49.3)	867 (41.8)	4444 (52.0)	
Female	323 (47.4)	2915 (50.7)	1206 (58.2)	4058 (48.0)	
Residence (%)					<0.001
Rural areas	647 (95.0)	2846 (49.5)	190 (9.2)	3683 (43.0)	
Urban city	34 (5.0)	2902 (50.5)	1883 (90.8)	4819 (57.0)	
Age Group (%)					<0.001
45–59	364 (53.5)	3158 (54.9)	1064 (51.3)	4492 (55.0)	
60–79	302 (44.3)	2352 (40.9)	933 (45.0)	3422 (42.0)	
80+	15 (2.2)	238 (4.1)	76 (3.7)	241 (3.0)	
Physical activity (%)					<0.001
High	373 (54.8)	1648 (28.7)	121 (5.8)	2142 (25.0)	
Moderate	179 (26.3)	1437 (25.0)	334 (16.1)	1950 (23.0)	
Low	129 (18.9)	2663 (46.3)	1618 (78.1)	4410 (52.0)	
Income level (%)					<0.001
Poverty	623 (91.5)	4397 (76.5)	1230 (59.3)	6250 (74.0)	
Non-poverty	44 (6.5)	1098 (19.1)	698 (33.7)	1840 (22.0)	
No response	14 (2.1)	253 (4.4)	145 (7.0)	412 (4.80)	
Smoke status (%)	204 (30.0)	1608 (28.0)	349 (16.8)	2161 (25.0)	<0.001
Drinking status (%)	148 (21.7)	1367 (23.8)	569 (27.4)	2084 (25.0)	<0.001
Education Group (%)					<0.001
Low	443 (65.1)	3298 (57.4)	695 (33.5)	4436 (52.0)	
Moderate	235 (34.5)	2239 (39.0)	1039 (50.1)	3513 (41.0)	
High	3 (0.4)	211 (3.7)	339 (16.4)	553 (6.5)	
Occupation Group (%)					<0.001
Business service	22 (3.2)	285 (5.0)	132 (6.4)	439 (5.2)	
Manual labor	373 (54.8)	1648 (28.7)	121 (5.8)	2142 (25.0)	
Office worker	17 (2.5)	453 (7.9)	239 (11.5)	709 (8.3)	
Others	31 (4.6)	775 (13.5)	225 (10.9)	1031 (12.0)	
Retired or unemployed	234 (34.4)	2498 (43.5)	1306 (63.0)	4038 (47.0)	
Student	4 (0.6)	89 (1.5)	50 (2.4)	143 (1.7)	
Overweight/obesity (%)	197 (28.9)	2809 (48.9)	1187 (57.3)	4193 (49.3)	<0.001
Hypertension (%)	324 (47.6)	3558 (61.9)	1412 (68.1)	5294 (62.0)	<0.001
Dyslipidemia (%)	11 (1.8)	595 (13.4)	534 (26.6)	1140 (16.0)	<0.001
Diabetes (%)	17 (2.7)	703 (14.8)	609 (30.2)	1329 (18.0)	<0.001

Continuous variables are presented as the mean ± SD, and categorical variables are presented as n (%) *p* values were derived from chi-square tests for categorical variables.

**Table 2:** Temporal transition of dietary patterns from 2002 to 2022.

Dietary Pattern	Linear OR (95%CI)	Linear *p*	Quadratic *p*	Joint Wald *p*	FDR-Adjusted *p*
Pattern 1	0.814 (0.773–0.857)	<0.001	<0.001	<0.001	<0.001
Pattern 2	0.980 (0.972–0.988)	<0.001	<0.001	<0.001	<0.001
Pattern 3	1.065 (1.055–1.074)	<0.001	<0.001	<0.001	<0.001

## Data Availability

The data presented in this study are available on request from the corresponding authors due to data management regulations of the National Institute for Nutrition and Health, Chinese Center for Disease Control and Prevention, which restrict public sharing of individual-level research data.

## Supplementary Materials

The following supporting information can be downloaded at https://www.mdpi.com/article/10.3390/nu18193222/s1. Figure S1: Elbow Method and Silhouette Coefficient for different cluster numbers; Figure S2: Principal component analysis visualization of the three dietary patterns; Figure S3: Distribution of dietary patterns across survey year; Table S1: Validity indices for k-means clustering across different numbers of clusters. Table S2: Food group intake across dietary patterns and the overall population; Table S3: Energy and nutrient intake across dietary patterns and the overall population; Table S4: Multivariable-adjusted associations between dietary patterns and overweight/obesity; Table S5: Multivariable-adjusted logistic regression analyses of dietary patterns in relation to overweight and obesity; Table S6: Subgroup and interaction analyses of the associations between dietary patterns and overweight/obesity; Table S7: Sensitivity analysis for overweight/obesity excluding participants with hypertension and diabetes; Table S8. Classification and representative foods of the 23 food groups used for dietary pattern analysis.

## Abbreviations

The following abbreviations are used in this manuscript:

BMI	Body mass index
CI	Confidence interval
CNNS	China National Nutrition Surveys
OR	Odds ratio
SD	Standard deviation
ARI	Adjusted Rand Index
CH	Calinski–Harabasz
DB	Davies–Bouldin
