# Supplementary material for: Temporal Transitions in Dietary Patterns and Their Associations with Overweight and Obesity Among Middle-Aged and Older Adults in Southern China: Evidence from Four Rounds of Nutrition Surveillance, 2002–2022
Source: Nutrients. 2026 Sep 29;18(19):3222. doi: 10.3390/nu18193222 (PMC13666665; doi:10.3390/nu18193222)
Supplement: Supplementary file 1 [file nutrients-18-03222-s001.zip › nutrients-4555664-supplementary.pdf]

Figure S1. Elbow Method and Silhouette Coefficient for different cluster numbers

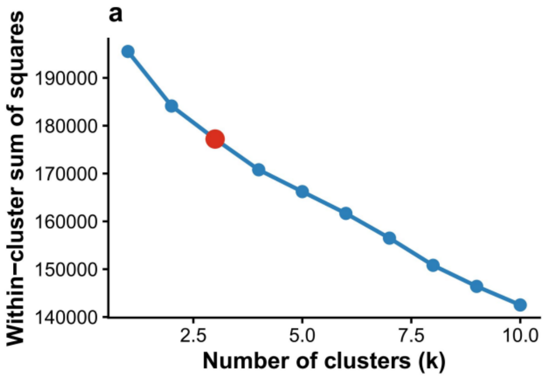

Table S1. Validity indices for k-means clustering across different numbers of clusters.

Method	k	Silhouette Coefficient	CH Index	DB Index	Dunn Index	Cluster sizes, n (%)
K-means	2	0.1849	526.3524	3.9696	0.0045	5896:2606
Hierarchical (ward.D2)	2	0.9076	346.8957	0.1560	0.5983	7965:537
Hierarchical (average)	2	0.9172	214.682	0.0682	0.5089	8501:1
K-means	3	0.1617	439.0823	3.4110	0.0032	5748:681:2073
Hierarchical (ward.D2)	3	0.1222	329.8933	1.9269	0.0088	7958:542:2
Hierarchical (average)	3	0.9076	289.8148	0.1256	0.6880	8499:2:1
K-means	4	0.1441	392.2788	3.5715	0.0037	661 ：1336 ：4898 ：1607
Hierarchical (ward.D2)	4	0.0623	311.1153	3.4232	0.0076	5109：2849:542:2
Hierarchical (average)	4	0.8596	275.8843	0.2878	0.2644	8496:2:3:1
K-means	5	0.1659	400.5204	2.4983	0.0041	1920:2:651:322:5607
Hierarchical (ward.D2)	5	0.0527	299.9209	2.9298	0.0076	5109:2849:538:2:4
Hierarchical (average)	5	0.8293	249.4692	0.2976	0.2931	8493:2:3:3:1

The silhouette coefficient, Calinski – Harabasz (CH) index, Davies – Bouldin (DB) index, Dunn index, and cluster sizes were calculated for candidate clustering solutions across k = 2 – 5. For the silhouette coefficient, CH index, and Dunn index, higher values indicate better clustering performance; for the DB index, lower values are preferable. The final three-cluster K-means solution was retained after considering internal validity indices, cluster size distribution, and nutritional interpretability.

Figure S2. Principal component analysis visualization of the three dietary patterns.

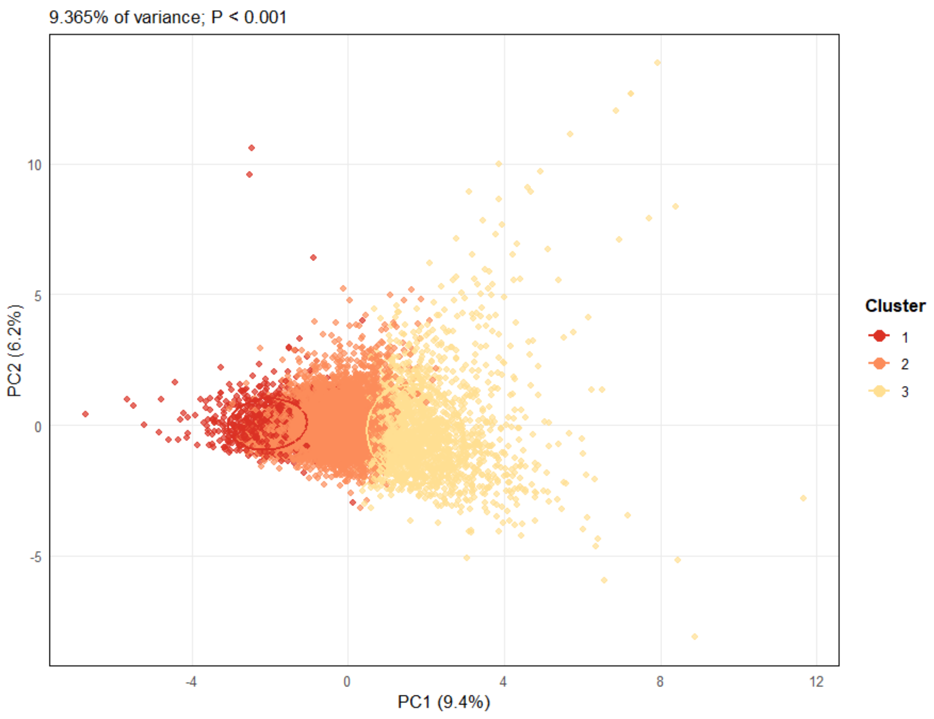

Table S2. Food group intake across dietary patterns and the overall population.

Food group	Dietary Clusters			Overall (n=8,502)	*P*_value
	Pattern 1 (n=681)	Pattern 2 (n=5,748)	Pattern 3 (n=2,073)		
Rice	384.82 ± 151.34	241.20 ± 129.56	161.99 ± 103.96	233.39 ± 137.61	< 0.001
Wheat	11.74 ± 29.69	32.24 ± 42.63	92.32 ± 81.67	45.25 ± 60.58	< 0.001
Other Cereals	0.55 ± 5.18	2.08 ± 8.78	17.41 ± 38.45	5.69 ± 21.43	< 0.001
Tubers	25.99 ± 85.18	11.83 ± 39.09	17.97 ± 43.28	14.46 ± 45.70	< 0.001
Fruit	30.40 ± 69.07	28.87 ± 54.29	125.83 ± 141.78	52.64 ± 94.88	< 0.001
Vegetables	340.96 ± 172.36	286.16 ± 145.80	426.94 ± 211.93	324.88 ± 176.83	< 0.001
Pork	97.05 ± 81.00	111.70 ± 82.33	128.56 ± 103.67	114.64 ± 88.35	< 0.001
Other Meat	3.54 ± 14.53	6.89 ± 21.07	22.71 ± 47.22	10.48 ± 30.16	< 0.001
Organ Meat	5.86 ± 23.53	4.56 ± 17.39	6.63 ± 21.16	5.17 ± 18.94	< 0.001
Poultry	20.70 ± 39.78	31.89 ± 47.40	63.99 ± 87.62	38.82 ± 61.07	< 0.001
Milk	0.38 ± 5.64	5.80 ± 26.93	84.08 ± 130.92	24.45 ± 76.28	< 0.001
Egg	14.07 ± 21.95	13.87 ± 20.72	43.14 ± 39.32	21.02 ± 29.38	< 0.001
Fish	33.11 ± 56.66	55.76 ± 67.74	97.85 ± 181.93	64.21 ± 108.75	< 0.001
Vegetable Oil	8.60 ± 15.96	35.95 ± 29.43	35.40 ± 28.99	33.62 ± 29.42	< 0.001
Animal Oil	40.71 ± 26.60	0.59 ± 2.81	0.18 ± 1.73	3.70 ± 13.49	< 0.001
Cake	1.19 ± 7.31	1.97 ± 10.39	14.24 ± 38.29	4.90 ± 21.51	< 0.001
Sugar	3.50 ± 10.86	3.96 ± 13.90	7.07 ± 19.98	4.68 ± 15.47	< 0.001
Salt	16.52 ± 39.92	8.93 ± 8.21	8.00 ± 8.46	9.31 ± 13.97	< 0.001
MSG	0.29 ± 1.83	0.36 ± 1.66	0.87 ± 10.85	0.48 ± 5.55	< 0.001
Sauce	7.87 ± 13.25	13.34 ± 24.32	18.79 ± 52.49	14.23 ± 33.08	< 0.001
Pastes	2.78 ± 10.18	2.37 ± 7.78	1.91 ± 7.69	2.29 ± 7.98	< 0.001
Alcohol	3.46 ± 18.14	3.85 ± 41.54	2.36 ± 27.12	3.46 ± 37.05	0.016
Soft Drinks	3.33 ± 20.90	1.39 ± 13.81	9.67 ± 51.97	3.57 ± 28.89	< 0.001

Data are presented as mean ± SD. Abbreviations: SD, standard deviation; MSG, monosodium glutamate. All values are expressed in grams per day (g/day) for a standard person. Statistical significance was defined as *P* < 0.05.

Table S3. Energy and nutrient intake across dietary patterns and the overall population.

Variable	Dietary Clusters			Overall (n=8,502)	P_ value
	Pattern 1 (n=681)	Pattern 2 (n=5,748)	Pattern 3 (n=2,073)		
Energy(kcal)	2542.56 ± 740.60	1981.05 ± 661.20	2459.53 ± 728.45	2142.69 ± 723.68	< 0.001
Protein(g)	76.38 ± 27.46	71.99 ± 35.64	107.00 ± 49.49	80.88 ± 41.68	< 0.001
Fat(g)	94.83 ± 44.60	83.08 ± 41.83	104.54 ± 47.10	89.26 ± 44.36	< 0.001
CHO(g)	344.79 ± 124.94	238.67 ± 105.13	279.85 ± 108.15	257.21 ± 111.99	< 0.001
Vitamin A (Retinol, μg)	498.38 ± 512.11	260.59 ± 381.18	387.05 ± 443.51	310.47 ± 416.04	< 0.001
Vitamin B1 (Thiamin, mg)	1.04 ± 0.43	0.90 ± 0.44	1.25 ± 0.54	1.00 ± 0.49	< 0.001
Vitamin B2 (Riboflavin, mg)	0.76 ± 0.30	0.79 ± 0.39	1.41 ± 0.77	0.94 ± 0.57	< 0.001
Vitamin B3 (Niacin, mg)	20.33 ± 7.01	19.20 ± 8.08	26.35 ± 13.70	21.04 ± 10.15	< 0.001
Vitamin E (Total, mg α-TE)	16.75 ± 11.91	23.69 ± 14.97	29.89 ± 17.46	24.65 ± 15.79	< 0.001
Vitamin E (α-Tocopherol, mg)	6.65 ± 3.98	9.58 ± 6.12	12.92 ± 7.19	10.16 ± 6.50	< 0.001
Vitamin E (β+γ-Tocopherol, mg)	4.06 ± 7.04	2.95 ± 5.57	3.22 ± 6.32	3.10 ± 5.90	< 0.001
Vitamin E (δ-Tocopherol, mg)	1.91 ± 3.11	1.02 ± 1.99	1.04 ± 2.12	1.10 ± 2.15	< 0.001
Sodium (mg)	8258.48 ± 15704.41	5233.81 ± 3914.12	5708.73 ± 5530.97	5591.88 ± 6180.70	< 0.001
Calcium (mg)	391.49 ± 172.99	391.76 ± 239.73	677.34 ± 381.53	461.37 ± 302.93	< 0.001
Magnesium (mg)	238.10 ± 106.34	242.65 ± 113.18	387.42 ± 174.38	277.58 ± 144.46	< 0.001
Iron (mg)	26.39 ± 23.06	21.35 ± 17.74	30.63 ± 20.43	24.01 ± 19.31	< 0.001
Manganese (mg)	6.16 ± 5.15	5.00 ± 5.64	6.76 ± 8.39	5.52 ± 6.43	< 0.001
Copper (mg)	8.09 ± 12.41	2.70 ± 5.20	2.54 ± 2.80	3.09 ± 5.89	< 0.001
Phosphorus (mg)	866.86 ± 327.62	899.10 ± 325.60	1409.13 ± 568.75	1020.87 ± 455.84	< 0.001
Selenium (μg)	51.13 ± 21.40	51.27 ± 26.46	75.90 ± 42.72	57.27 ± 32.66	0.016
Zinc (mg)	12.39 ± 4.96	12.13 ± 5.47	16.67 ± 10.03	13.25 ± 7.10	< 0.001

Data are presented as mean ± SD. Abbreviations: SD, standard deviation. All intakes are expressed per day for a standard person. Statistical significance was defined as *P* < 0.05.

Figure S3. Distribution of dietary patterns across survey year

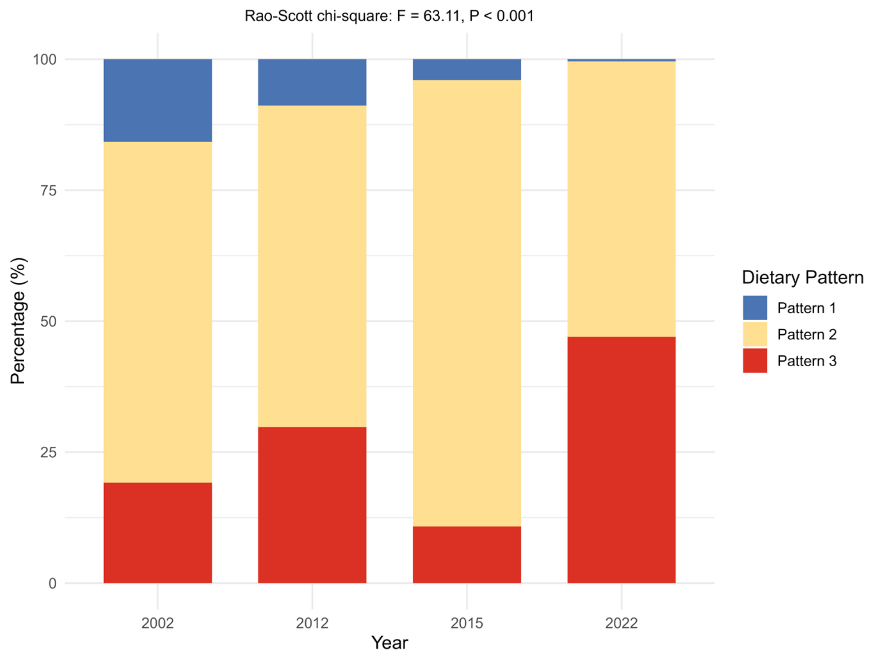

Distribution of dietary patterns across survey years. The figure shows the proportion of participants classified into each dietary pattern across the four survey waves (2002, 2012, 2015, and 2022). Differences in the distribution of dietary patterns across survey waves were statistically significant (Rao − Scott adjusted chi-square test, P < 0.001).

Table S4. Multivariable-adjusted associations between dietary patterns and overweight/obesity

Pattern	Model	OR(95% CI)	P value
Pattern 2	Model 1	2.35(1.98 – 2.80)	<0.001
	Model 2	1.66(1.38 – 2.00)	<0.001
	Model 3	1.58(1.31 – 1.90)	<0.001
	Model 4	1.57(1.29 – 1.90)	<0.001
Pattern 3	Model 1	3.29(2.73 – 3.98)	<0.001
	Model 2	1.74(1.40 – 2.15)	<0.001
	Model 3	1.53(1.23 – 1.91)	<0.001
	Model 4	1.42(1.14 – 1.77)	0.002

Multivariable logistic regression models were used to estimate odds ratios (ORs) and 95% confidence intervals (CIs) for overweight/obesity (n = 8,502).
Pattern 1 was used as the reference group.
Model 1 was unadjusted.
Model 2 was adjusted for age, sex, survey year, and region.
Model 3 was further adjusted for education level and income level.
Model 4 was additionally adjusted for smoking status, alcohol consumption, physical activity, and total energy intake.
Statistical significance was defined as $P < 0.05$.

Table S5. Multivariable-adjusted logistic regression analyses of dietary patterns in relation to overweight and obesity

Multivariable logistic regression models were used to estimate odds ratios (ORs) and 95% confidence intervals (CIs) for overweight ,obesity and central obesity (n = 8,502).
Pattern 1 was used as the reference group.
Model 1 was unadjusted.

Outcome	Pattern	Model	OR（95% CI）	*P* value
Obesity	Pattern 2	Model 1	2.62 (2.08 – 3.29)	<0.001
		Model 2	1.84 (1.45–2.32)	<0.001
		Model 3	1.74 (1.36–2.23)	<0.001
		Model 4	1.73 (1.35–2.23)	<0.001
	Pattern 3	Model 1	3.63 (2.84–4.64)	<0.001
		Model 2	1.91 (1.48–2.47)	<0.001
		Model 3	1.73(1.31–2.29)	<0.001
		Model 4	1.58 (1.19–2.10)	0.001
Overweight	Pattern 2	Model 1	2.11 (1.68–2.62)	<0.001
		Model 2	1.48 (1.19–1.86)	<0.001
		Model 3	1.42 (1.12–1.80)	<0.001
		Model 4	1.41(1.11–1.80)	0.005
	Pattern 3	Model 1	2.98 (2.36–3.78)	<0.001
		Model 2	1.57 (1.22–2.01)	<0.001
		Model 3	1.35 (1.03–1.78)	<0.001
		Model 4	1.26 (0.96–1.66)	0.098
Central obesity	Pattern 2	Model 1	2.27(1.89–2.73)	<0.001
		Model 2	1.64(1.35–1.99)	<0.001
		Model 3	1.55(1.27–1.89)	<0.001
		Model 4	1.54(1.26–1.89)	<0.001
	Pattern 3	Model 1	2.94(2.42–3.57)	<0.001
		Model 2	1.62(1.30–2.03)	<0.001
		Model 3	1.44(1.15–1.81)	0.002
		Model 4	1.35(1.08–1.71)	0.010

Model 2 was adjusted for age, sex, survey year, and region.
Model 3 was further adjusted for education level and income level.
Model 4 was additionally adjusted for smoking status, alcohol consumption, physical activity, and total energy intake.
Statistical significance was defined as *P* < 0.05.

Table S6. Subgroup and interaction analyses of the associations between dietary patterns and overweight/obesity.

Subgroup	Pattern 2		Pattern 3		*P* for interaction
	Adjusted OR (95% CI)	*P* value	Adjusted OR (95% CI)	*P* value	
**Year**					0.013
2002	1.33 (0.99 — 1.79)	0.064	1.02 (0.70 — 1.48)	0.932	
2012	1.74 (1.19 — 2.56)	0.004	1.79 (1.17 — 2.74)	0.007	
2015	1.10 (0.72 — 1.68)	0.654	1.02 (0.61 — 1.70)	0.946	
2022	1.58 (0.29 — 8.72)	0.579	1.41 (0.25 — 7.85)	0.678	
**Residence**					0.239
Rural areas	1.32 (1.07 — 1.63)	0.011	1.51 (1.03 — 2.21)	0.034	
Urban city	1.68 (0.84 — 3.36)	0.139	1.50 (0.75 — 3.01)	0.254	
**Sex**					0.005
Female	1.58 (1.21 — 2.07)	<0.001	1.40 (1.03 — 1.90)	0.031	
Male	1.51 (1.14 — 2.00)	0.004	1.51 (1.09 — 2.09)	0.014	
**Age**					0.612
45–59	1.58 (1.22 — 2.06)	<0.001	1.37 (1.01 — 1.85)	0.043	
60–79	1.52 (1.14 — 2.05)	0.043	1.43 (1.02 — 2.02)	0.037	
80+	1.93 (0.51 — 9.54)	0.367	2.27 (0.49 — 12.86)	0.314	

Pattern 1 was used as the reference group.
Models were adjusted for age, sex, education level, income level, smoking status, alcohol consumption, physical activity, total energy intake, survey year, and region.
P - values were derived from multivariable logistic regression models.
P for interaction was calculated using likelihood ratio tests.
Statistical significance was defined as *P* < 0.05.

Table S7. Sensitivity analysis for overweight/obesity excluding participants with hypertension, diabetes or dyslipidemia

**Pattern**	Model	OR(95% CI)	*P* value
Pattern 2	Model 1	1.83 (1.37 — 2.5)	<0.001
	Model 2	1.37 (0.99 —1.91)	0.060
	Model 3	1.34 (0.97 —1.88)	0.079
	Model 4	1.33 (0.95 — 1.87)	0.102
Pattern 3	Model 1	2.49 (1.77 — 3.54)	<0.001
	Model 2	1.42 (0.93 — 2.16)	0.104
	Model 3	1.31 (0.86 — 2.01)	0.218
	Model 4	1.17 (0.76 — 1.81)	0.474

Multivariable logistic regression models were used to estimate odds ratios (ORs) and 95% confidence intervals (CIs) for overweight/obesity. Participants with hypertension, diabetes or dyslipidemia were excluded from this analysis (n = 2082).
Pattern 1 was used as the reference group.
Model 1 was unadjusted.
Model 2 was adjusted for age, sex, survey year, and region.
Model 3 was further adjusted for education level and income level.
Model 4 was additionally adjusted for smoking status, alcohol consumption, physical activity, and total energy intake.
Statistical significance was defined as $P < 0.05$.

Table S8. Classification and representative foods of the 23 food groups used for dietary pattern analysis.

No.	Food categories	Representative foods included
1	Rice	Japonica rice, indica rice, glutinous rice, brown rice, black and other colored rice, cooked rice, rice porridge, rice noodles and rice vermicelli, rice flour, and other rice products
2	Wheat	Wheat and wheat flour, wheat bran and germ, noodles and pasta, steamed bread and rolls, wheat-based flatbreads and fried dough products, wheat gluten, bread, and other wheat-based products
3	Other Cereals	Maize, barley and hulless barley, millet and proso millet, sorghum, buckwheat and tartary buckwheat, oats, coix seed, quinoa, cereal flakes and porridges, and other miscellaneous cereal products
4	Tubers	Potatoes, sweet potatoes, cassava, potato and sweet potato flour, and other tuber products
5	Fruit	Apples, pears, peaches, plums, apricots, dates, cherries, grapes, pomegranates, persimmons, berries, kiwifruit, citrus fruits, bananas, pineapples, lychees, longans, mangoes, papayas, guavas, dragon fruit, melons, watermelons, and other fruits
6	Vegetables	Radishes, carrots and other root vegetables; fresh beans, peas and sprouts; tomatoes, eggplants and peppers; cucumbers, gourds and squashes; leafy vegetables; allium vegetables; stem and flower vegetables; fungi and algae; and other vegetables
7	Pork	Fresh pork and pork cuts, pork ribs, trotters and skin, roasted and braised pork, cured and preserved pork, ham, sausages, luncheon meat, pork floss, and other pork products
8	Other Meat	Beef, mutton and lamb, donkey meat, camel meat, rabbit meat, venison, and their cooked or processed products
9	Organ Meat	Liver, kidney, heart, lung, stomach and tripe, intestine, brain, tongue, blood, gizzard, and other edible organs from livestock and poultry
10	Poultry	Chicken, duck, goose, turkey, pigeon, quail, and their cooked or processed products
11	Milk	Cow's milk, goat's milk, milk powder and formula milk, yogurt and other fermented milk products, cheese, and other dairy products
12	Egg	Chicken eggs, duck eggs, goose eggs, quail eggs, egg white and yolk, preserved and salted eggs, and other egg products
13	Fish	Fish, shrimp,shellfish, and other aquatic foods
14	Vegetable Oil	Soybean oil, rapeseed oil, salad oil, sunflower seed oil, and other vegetable oils
15	Animal Oil	Lard, beef tallow, and other animal fats
16	Cake	Cakes, chocolate, cookies, pastries, and other bakery and confectionery products
17	Sugar	White sugar, brown sugar, and other sugars
18	Salt	Cooking salt
19	MSG	Monosodium glutamate (MSG), chicken essence, seasoning powders, and other compound seasonings
20	Sauce	Soy sauce, including light soy sauce, dark soy sauce, and other varieties of soy sauce
21	Pastes	Vinegar, fermented bean pastes, chili sauces, sesame and peanut pastes, salad dressings, fruit preserves, tomato ketchup, oyster sauce, fermented bean curd, and other sauces and pastes
22	Alcohol	Beer, wine, Chinese rice wine, Chinese spirits (baijiu), fruit wine, brandy, whisky, vodka, cocktails, and other alcoholic beverages
23	Soft Drinks	Carbonated soft drinks, fruit juices and fruit-flavored drinks, sports drinks, milk-based and plant-based beverages, tea and coffee beverages, solid beverage mixes, and other non-alcoholic beverages

Representative foods are shown for each food group and do not constitute an exhaustive list of all individual food items or food codes. Individual food items were identified and classified according to the CNNS food coding system and the Chinese Food Composition Tables. Where applicable, recorded food weights were standardized to a comparable edible-weight basis using appropriate food-specific conversion factors, such as edible-portion coefficients and raw-to-cooked or cooked-to-raw weight conversion factors. Other food-specific weight conversions were applied as required according to the form in which foods were recorded. Standardized food weights assigned to the same food group were aggregated to calculate individual daily food-group intakes. The same food-group classification framework was applied across survey waves to ensure comparability.
